# Novel Class-AB Operational Amplifier for Compact and Energy-Efficient Wake-Up Sensor Systems

**DOI:** 10.3390/s25020316

**Published:** 2025-01-07

**Authors:** Hussam AlShammary

**Affiliations:** 1 Electrical Engineering Department, King Fahd University of Petroleum and Minerals (KFUPM), Dhahran 31261, Saudi Arabia; hussamsh@kfupm.edu.sa; 2 Center for Communication Systems and Sensing, King Fahd University of Petroleum and Minerals (KFUPM), Dhahran 31261, Saudi Arabia

**Keywords:** Class-AB operational amplifier, energy-efficient sensors, low-power design, Internet of things (IoT), rail-to-rail, energy-efficient circuits, electronic sensors

## Abstract

This paper presents a novel rail-to-rail Class-AB operational amplifier tailored for wake-up systems in motion sensor applications. By addressing limitations in free Class-AB designs, such as large inrush current, unstable bias conditions, and area ineffiiency, the proposed design achieves a gain of 59 dB and unity gain frequency of 550 kHz driving a 5 pF load. The inrush current is reduced from 1 mA to 7 µA, increasing the battery life. The layout area is reduced by 53% compared to the free Class-AB design, making it highly suitable for compact implementations. Operating at a low power consumption of 2 µW with a 1.8 V supply, the amplifier achieves a Signal-to-Noise-and-Distortion Ratio (SNDR) of 22 dB. These advancements demonstrate the potential of the design for energy-efficient analog front-end solutions in IoT and portable systems.

## 1. Introduction

Modern low-power systems, such as motion sensors in IoT and wearable applications, rely on energy-efficient analog front-end circuits (AFEs) to process small signals accurately. Wake-up functionality plays a crucial role in these systems by allowing devices to remain in low-power sleep modes and activate only when triggered by specific events. This significantly extends battery life while maintaining reliable performance. Motion sensors, such as Micro-Electro-Mechanical System (MEMS)-based accelerometers, produce low-amplitude signals (0.5 mV to 10 mV) that require precise amplification to interface with analog-to-digital converters (ADC). These systems impose stringent specifications on AFEs, including low noise, robust Common-Mode Rejection Ratio (CMRR) and Power Supply Rejection Ratio (PSRR), minimal distortion, and fast response times. Table 1 summarizes the key specifications for AFEs tailored to motion sensor applications, highlighting the challenges these systems face in achieving compactness, low power, and high performance levels. These requirements, particularly the need for low inrush current and efficient operation during wake-up transitions, have motivated advancements in Class-AB amplifier designs.

While Class-A operational amplifiers (op-amp) provide low inrush current, they lack a decent signal-to-distortion and noise ratio (SNDR) due to their poor slew rate and limited current availability during rapid transitions. While versatile, traditional Class-AB [1] designs encounter several challenges, including their excessive area, startup current surges, biasing instability, and linearity issues. Recent research efforts have aimed at addressing these limitations with innovative techniques. An effective Class-AB design leveraging rail-to-rail operation with high linearity and reduced area is presented in [2]. This approach improves efficiency while achieving high output current, making it ideal for portable systems. While its SNDR improved by several folds compared to Class-A op-amp, large inrush current and unstable bias voltage can significantly drain the battery.

Significant advancements in Class-AB operational amplifier design have addressed various challenges in compactness, power efficiency, and performance. Techniques for improving gain bandwidth and stability have been explored extensively. For example, auxiliary circuit enhancements [3], nested Miller compensation [4], and low-voltage design innovations [5] have improved transconductance, gain, and stability across a wide range of capacitive loads. Additionally, local common-mode feedback [6] and adaptive biasing schemes [7] have demonstrated the feasibility of energy-efficient amplifiers compatible with sub-1 V supplies.

Other works have focused on optimizing Class-AB designs for specific applications. Rail-to-rail voltage conveyors [8] have enhanced the versatility of Class-AB amplifiers by supporting wider input ranges, while tunable gain architectures [9] have allowed for adaptability in performance across various applications. Capacitive-feedback amplifiers [10] have improved noise suppression, and nonlinear nested current mirrors [11] have addressed distortion performance effectively. Low-power Class-AB OTAs [12,13] and three-stage LDOs [14] have been developed with a focus on portable and biomedical systems, emphasizing area efficiency and low quiescent current.

Despite these advancements, significant gaps remain in adapting Class-AB designs to the unique demands of wake-up systems and motion sensors. Earlier designs, such as free Class-AB op-amps, suffer from large inrush currents during transitions, undefined bias conditions, and inefficient area utilization. For example, compact rail-to-rail amplifiers [15,16] demonstrate versatility in oscillator-based systems but lack tailored solutions for dynamic wake-up scenarios. Similarly, baseband amplifiers [17,18,19] optimized for RF front ends are not directly applicable to low-power motion sensing; however, they can benefit from the proposed Class-AB technique.

The performance specifications in Table 1, including bandwidth, input signal levels, and noise, are influenced by recent advancements in MEMS interface circuits and modeling. Closed-loop designs with correlated double sampling [20] informed the stringent noise requirements, ensuring compatibility with low-amplitude signals common in motion sensors. Similarly, frequency-based readout circuits [21] highlighted the importance of balancing bandwidth and power consumption. The choice of a 10 kHz bandwidth reflects a design aimed at supporting both low-frequency applications, such as passive infrared (PIR) sensors, and higher-frequency use cases, like vibration analysis with MEMS accelerometers, ensuring flexibility across a wide range of motion sensing applications.

This paper compares a traditional Class-A to free Class-AB [2] and the proposed Class-AB tailored for the specifications in Table 1. Section 2 is dedicated to circuit analysis, while Section 3 goes over the design details in TSMC 180 nm. Simulation results are discussed in Section 4. A comparison against state-of-the-art work on Class-AB for different applications is made through two figures of merits (FOMs).

## 2. Circuit Analysis of the Proposed Class-AB Op-Amp

**Figure 1:**
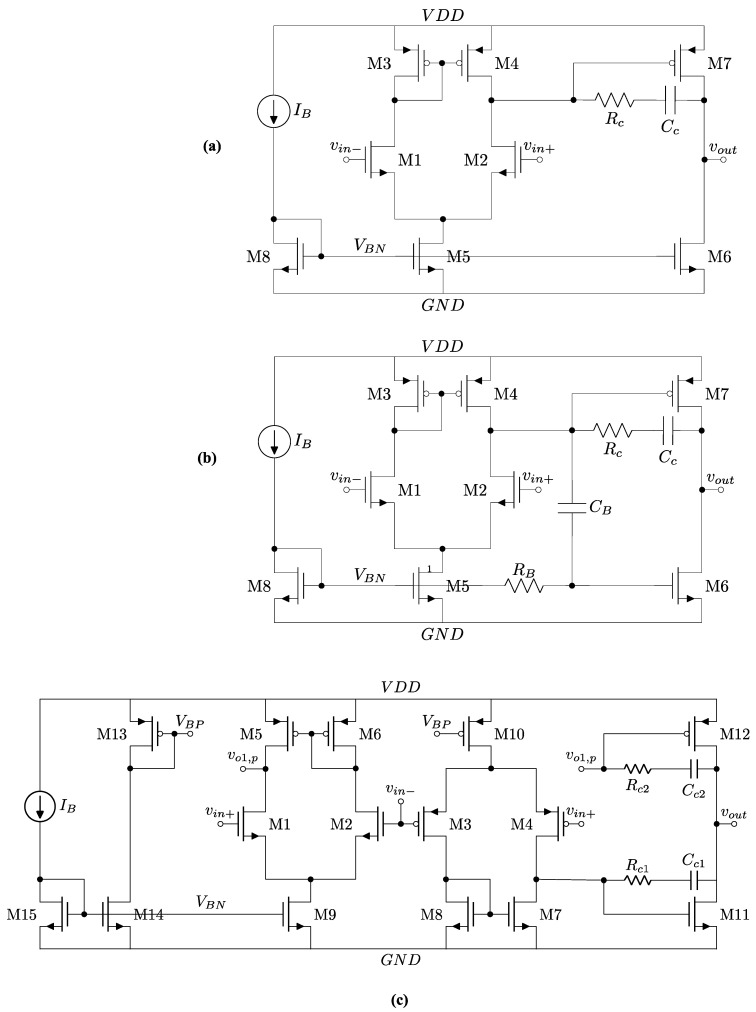
(**a**) Class-A op-amp with Miller compensation; (**b**) free Class-AB op-amp based on the addition of C_B_ and R_B_ [2]; (**c**) proposed Class-AB op-amp with complementary input stage.

Figure 1a shows a circuit-level diagram for a generic Class-A (Miller) op-amp. The output stage driving capability is limited in the negative direction by the fixed current of M6. Figure 1b illustrates a free Class-AB [2] by tapping the output of the first stage and DC coupling it to the second stage through the gate of M6, which serves the purpose of a current source and a transconductance stage, unlike in Class-A, where it serves as a current source only. The low-frequency gain of a Class-A op-amp is as follows:

A_A_ = g_m1_ (r_o2_||r_o4_) g_m7_(r_o6_||r_o7_), (1)

while the low-frequency gain of a free Class-AB op-amp is higher due to added path through M6:

A_AB,free_ = g_m1_ (r_o2_||r_o4_) (g_m6_ + g_m7_) (r_o6_||r_o7_). (2)

The gains of Class-A and free Class-AB start the same at low frequencies and equal to A_A_, then the gain increases for Class-AB only at the zero-frequency, given by the following:

(3) $$f_{Z}=\frac{1}{2\pi {R_{B}C}_{B}}.$$

While the extra path provides several advantages such as an improved slew rate in the negative cycle, it poses a risk during start-up, as the gate of M6 remains floating, as will be discussed in detail in the simulation section. Furthermore, the addition of C_B_ increases the area of the op-amp significantly.

Figure 1c shows the proposed Class-AB. It relies on two complementary input stages and is directly connected to the output stage without the explicit need for R_B_ and C_B_. The low-frequency gain of the proposed Class-AB can be found as follows:

(4) $$\begin{array}{cc} A_{\mathrm{AB},\mathrm{proposed}} & =g_{m1}\;(r_{o2}||r_{o6})\;g_{m12}\;(r_{o11}||r_{o12})\;+\;g_{m3}\;(r_{o4}||r_{o7})\;g_{m11}\;(r_{o11}||r_{o12})\\ & =\;\;\;\;\;\;A_{1}\;\;\;\;\;\;\;\;+\;\;\;\;\;\;A_{2}\\ & =\;g_{m,n}\;(r_{o,n}||r_{o,p})\;g_{m,p}\;(r_{o,n}||r_{o,p})\;+\;g_{m,p}\;(r_{o,p}||r_{o,n})\;g_{m,n}\;(r_{o,n}||r_{o,p}), \end{array}$$

which is about twice the gain of Class-A down to DC, unlike free Class-AB. To compensate the Class-AB op-amp, two Miller capacitors are used with poles for each path:

(5) $$\omega_{P1}=\frac{1}{{g_{m12}(r_{o11}||r_{o12})(r_{o2}||r_{o6})C}_{c1}},$$

(6) $$\omega_{P2}=\frac{1}{{g_{m11}(r_{o11}||r_{o12})(r_{o4}||r_{o7})C}_{c2}}.$$

The high-frequency gain of the proposed Class-AB can be found as follows:

(7) $$A_{AB,\;proposed}=\frac{A_{1}}{1+{\frac{S}{\omega }}_{P1}}+\frac{A_{2}}{1+{\frac{S}{\omega }}_{P2}}\;=(A_{1}+A_{2})\frac{\left(1+S\frac{{\frac{A_{1}}{\omega }}_{P1}+{\frac{A_{2}}{\omega }}_{P2}}{A_{1}+A_{2}}\right)}{\left(1+{\frac{S}{\omega }}_{P1}\right)\left(1+{\frac{S}{\omega }}_{P2}\right)}$$

which is a second-order system with two poles and one zero. Assuming the gains A_1_ and A_2_ are about the same, we obtain an approximate expression for the feedforward zero:

(8) $$f_{Z,FF}=\frac{f_{P1}+f_{P2}}{2}.$$

The zero and the second pole phases cancel each other at frequencies much higher than the second pole. The pole f_p2_ is the dominant pole since g_m11_ > g_m12_ (NMOS has a higher g_m_/I_D_ than PMOS).

## 3. Proposed Design Details

**Figure 2:**
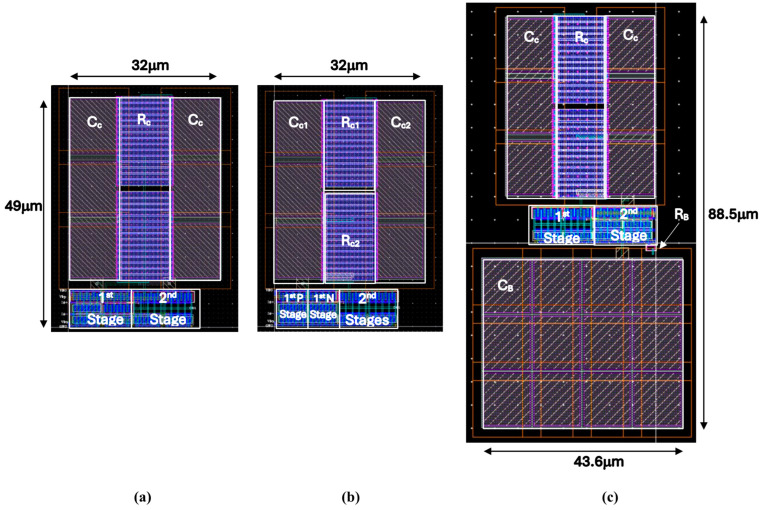
Layouts in TSMC 180 nm of (**a**) Class-A op-amp; (**b**) proposed Class-AB op-amp; (**c**) free Class-AB op-amp.

The three different topologies in Figure 1 are simulated in TSMC 180 nm process. Table 2 shows the values used in the design, including I_B_ = 0.25 μA and C_L_ = 5 pF. Figure 2 shows the layout of the three topologies. The proposed’s Class-AB op-amp layout is similar to Class-A and occupies less than half the area of free Class-AB.

## 4. Simulation Results and Discussion

**Figure 3:**
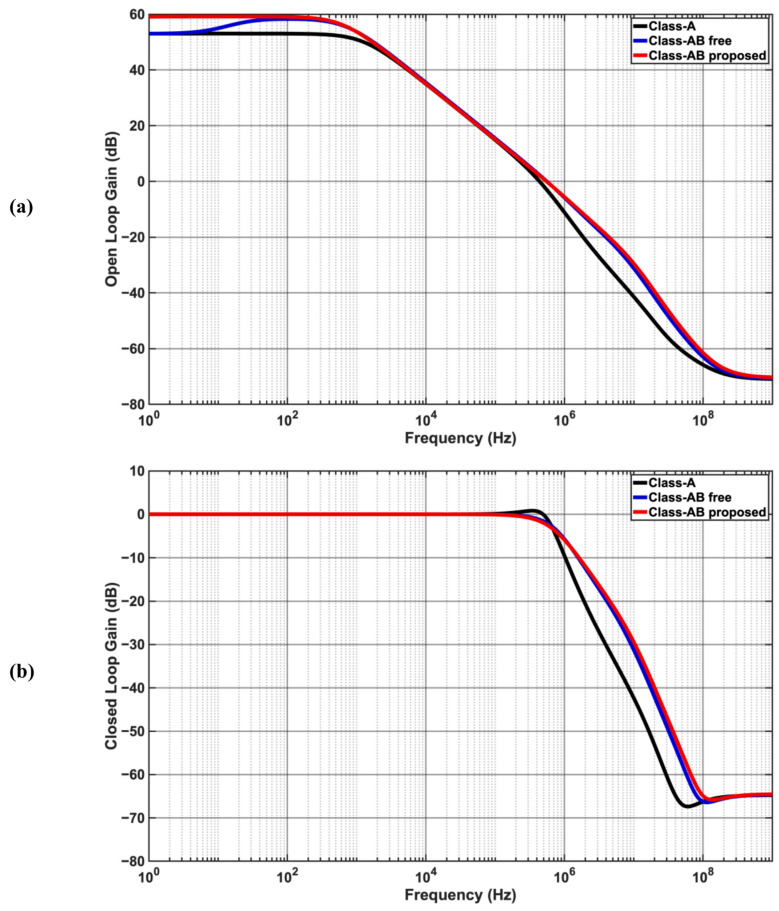
Frequency response showing improved flatness of gain in the proposed Class-AB design: (**a**) open-loop small-signal gain; (**b**) closed-loop small signal gain.

Figure 3a shows the simulated small-signal open-loop gains for all topologies vs. frequency. The open-loop gain at low frequencies is around 53 dB for the Class-A op-amp. It is observed for the free Class-AB op-amp that the gain increases at frequencies higher than approx. 8 Hz. The extra gain is due to the added path of M6, which is approx. 6 dB. Unlike the free Class-AB op-amp, the low-frequency gain of the proposed Class-AB remains flat at about 59 dB, which is approximately twice the gain of the Class-A design. The input and pad capacitances are crucial, as they load the motion sensor. The estimated total input capacitance from simulation are approx. 0.25 pF for each design, satisfying the requirement in Table 1.

Figure 3b shows the simulated small signal closed loop with unity gain for all topologies vs. frequency, showing a 3 dB Bandwidth (BW) of around 600 kHz. Table 3 summarizes the small signal parameters of the three topologies. A reduction in PSRR is observed in the free and proposed Class-AB due to the change to the output stage (M12) where the cancellation is less. Nevertheless, the simulated PSRR meets the requirement of Table 1. 

The frequency response of CMRR is dominated by the zero at the virtual ground nodes of each stage (drains of M5 in Class-A and free Class-AB and M9 and M10 of proposed Class-AB), thus limiting the bandwidth of common mode rejection. CMRR and PSRR 3 dB BW as well as their worst values are reported in Table 3. 

The large signal simulation includes a two-tone test applied at f_1_ and f_1_ + 1 kHz (fundamentals) with a total power of +4 dBm (1 Vpp), while the third-order intermodulation (IM3) product is observed at f_1_ − 1 kHz in dBc referred to the power of fundamentals. f_1_ is swept from 25 kHz to 500 kHz in steps of 25 kHz. Similarly, the second-order intermodulation (IM2) is observed at 1 kHz in dBc referred to the power of fundamentals.

**Figure 4:**
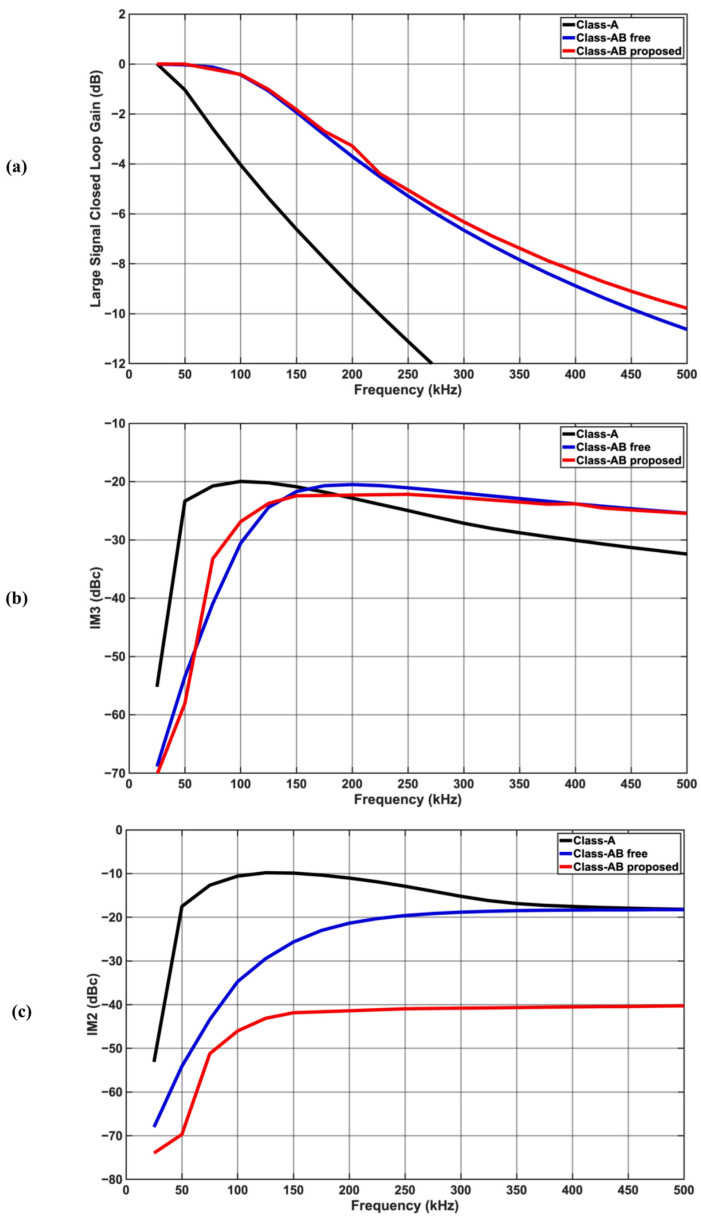
Large signal frequency response in a two-tone test with 1 Vpp. (**a**) Closed-loop gains showing poor Class-A BW; (**b**) third-order intermodulation (IM3) distortion in dBc; (**c**) second-order intermodulation (IM2) distortion in dBc with superior performance for proposed Class-AB.

Figure 4a shows the large signal gain at different frequences. The Class-A 3 dB BW is reduced to 80 kHz due to the slew rate limitation of the topology. The Class-AB op-amps show more resilience to large signal operation, and their 3 dB BW is approx. 175 kHz. Figure 4b plots the IM3 component against different frequencies. Within the 3 dB BW, the worst IM3 component is −22.4 dBc for the proposed Class-AB compared to −20.7 dBc for free Class-AB. While Class-A shows similar worst IM3 numbers of −20.7 dBc, its 3 dB BW is much narrower. IM3 is a crucial metric that captures in band spurs generated by the modulated signal that can dominate the noise floor. Another mechanism is IM2, plotted in Figure 4c against different frequencies. Within the 3 dB BW, the worst IM2 component is −12.4 dBc, −29.5 dBc, and −43.3 dBc for Class-A, free Class-AB, and the proposed Class-AB, respectively, which shows approx. 14 dB improvement in the proposed Class-AB due to the complementary input stage that shows flat gain against varying input levels.

**Figure 5:**
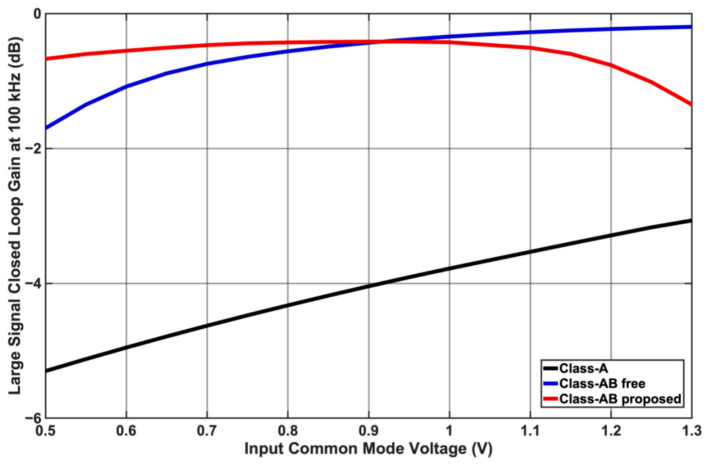
Large signal gain for each topology at 100 kHz input frequency vs. input common mode bias voltage showing better flatness for the proposed Class-AB.

**Figure 6:**
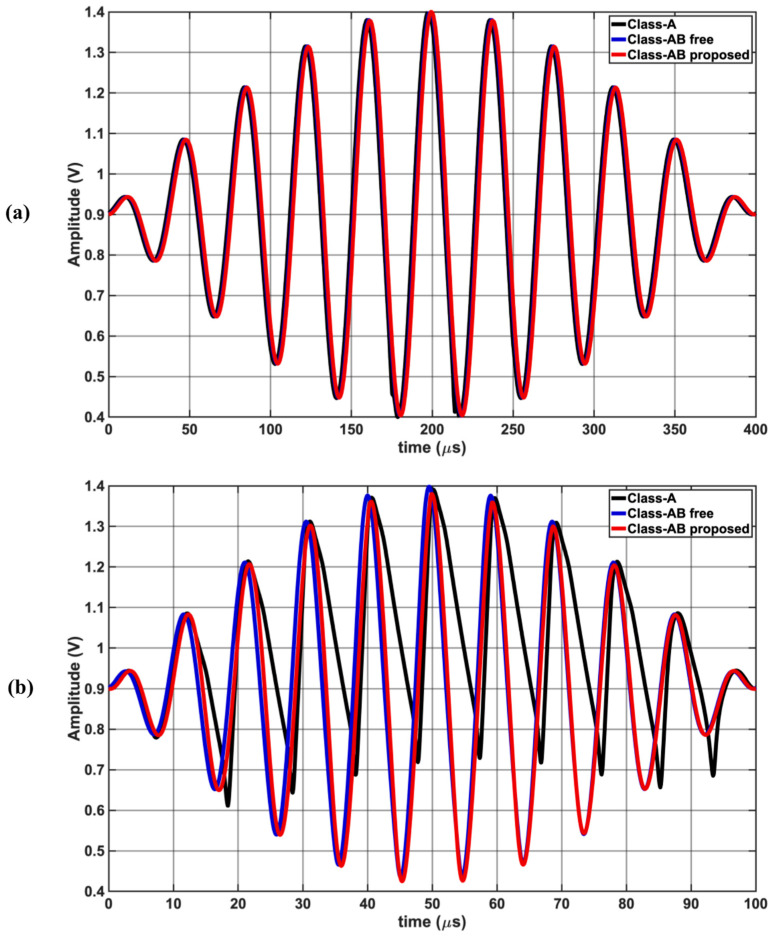
Time domain outputs of all three op-amps in closed-loop configuration in a two-tone test with 1 Vpp (IM3) at (**a**) f_1_ = 25 kHz, (**b**) f_1_ = 100 kHz.

**Figure 7:**
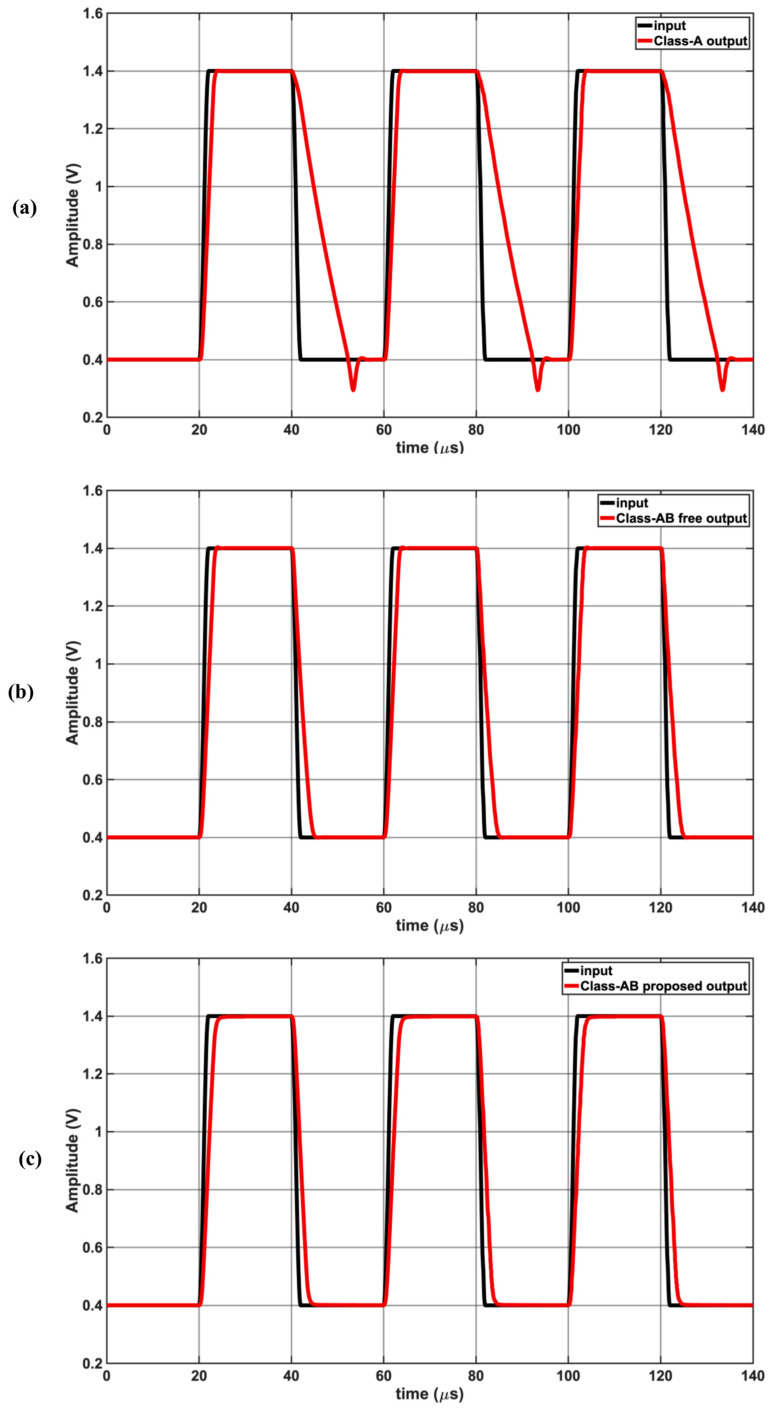
Time domain 1 Vpp pulse response input and output waveforms at 25 kHz: (**a**) Class-A with poor slew rate; (**b**) free Class-AB; (**c**) proposed Class-AB.

Figure 5 compares the large signal gain of a 1 Vpp signal at 100 kHz as the input common mode bias voltage is varied from 0.5 V to 1.3 V, demonstrating rail-to-rail operating. The proposed Class-AB has the flattest response, allowing for rail-to-rail operation. Figure 6 shows the time domain output waveforms for each op-amp when the input is two tones at (a) 25 kHz and 26 kHz (b) 100 kHz and 101 kHz. Class-A struggles to keep up with the 100 kHz and 101 kHz tones due to the limited slew rate. Figure 7 shows the simulated input and output waveforms of the op-amps for a 25 kHz 1 Vpp input square waveform. The corresponding measured slew rates were 0.081 V/μs for Class-A and approx. 0.33 V/μs for the proposed Class-AB.

**Figure 8:**
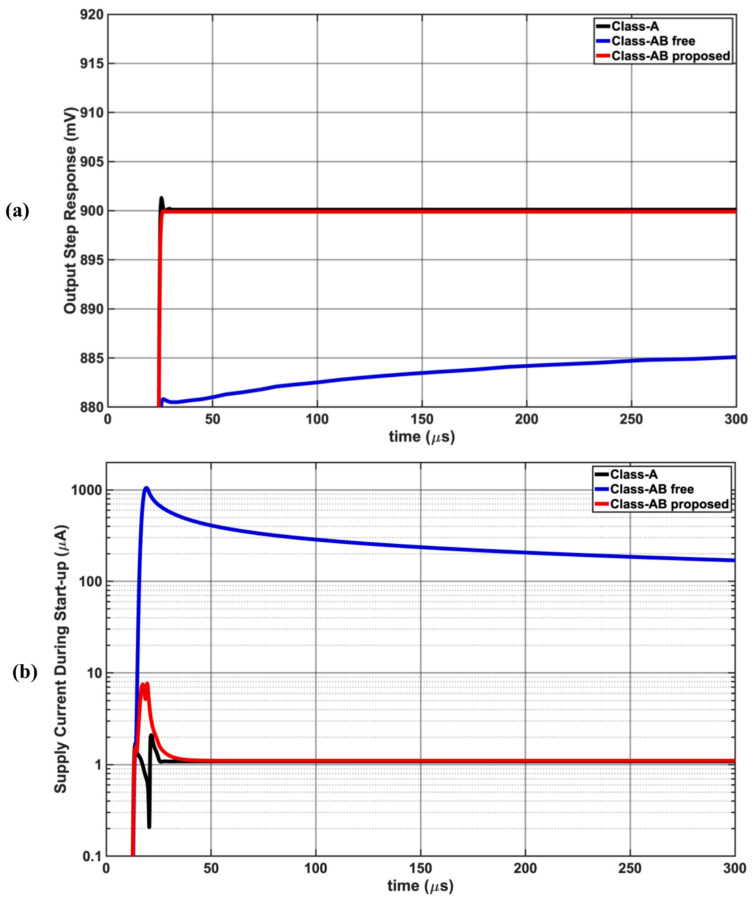
Start-up dynamics: (**a**) steady-state step response showing larger error for free Class-AB op-amp; (**b**) inrush and steady-state currents for all 3 stages with poor free Class-AB inrush.

Figure 8 shows the start-up dynamics of the three op-amps with supply ramp-up. A steady-state error of 15 mV at 300 μs for free Class-AB is observed, while it remains under 0.1 mV for Class-A and the proposed Class-AB. Also, a large inrush current of above 1 mA is observed for free Class-AB, and the current does not approach 1 μA, which it was designed for. The proposed Class-AB has a lower inrush current of 7 μA but settles quickly to the desired value of 1 μA. A large generated inrush current every time the op-amp is enabled to listen for possible activity is power-hungry, preventing the wake-up feature.

Table 4 summarizes the simulated large signal parameters of the three topologies for motion sensor applications. The proposed Class-AB op-amp shows a 2.3 dB improvement in SNDR. All three topologies are second-order distortion limited. The SNDR is estimated based on noise, and the IM3 and IM2 distortions (all in scalar):

(9) $$SNDR=\frac{1}{\frac{1}{SNR}+{IM}_{3}+IM_{2}}.$$

**Figure 9:**
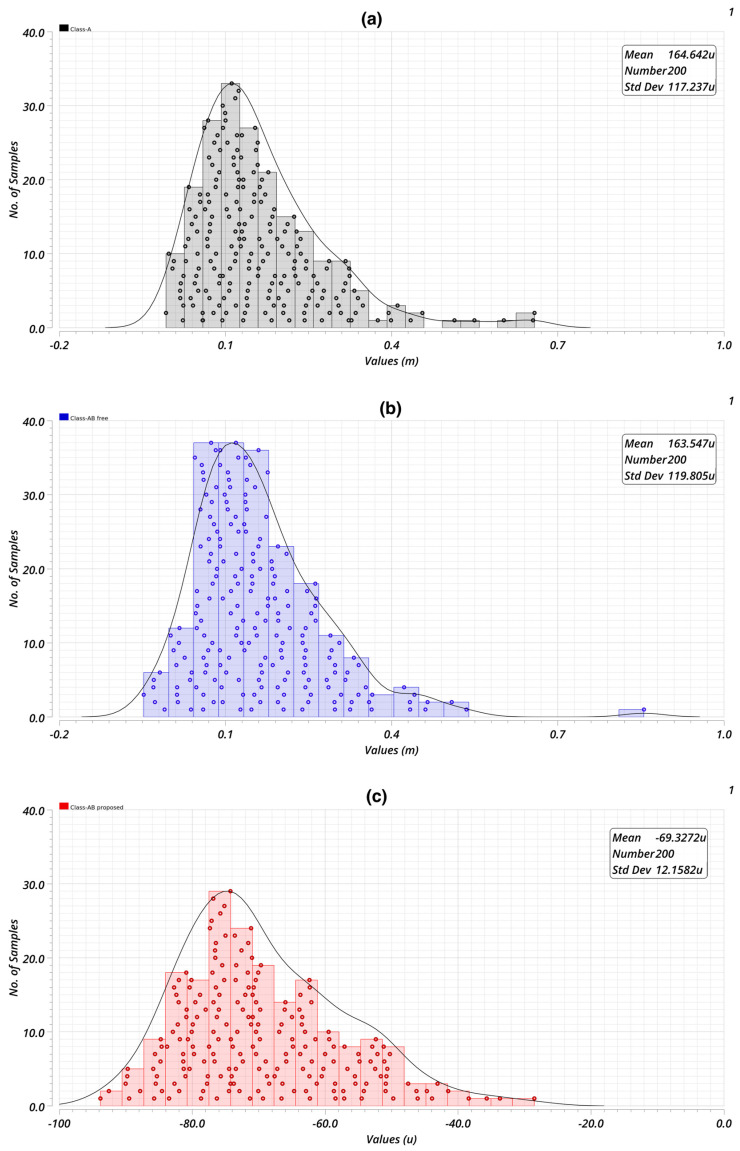
Input offset voltage due to mismatch with 200 pt Monte-Carlo simulation: (**a**) Class-a with 0.165 mV mean; (**b**) Class-ab free with 0.165 mV mean; (**c**) proposed Class-ab with −0.070 mV mean.

In the context of conventional Class-A and free Class-AB amplifiers, the input offset voltage due to mismatch is a critical factor affecting performance. Both of these architectures often rely on single-polarity input stages (e.g., NMOS or PMOS), where mismatch arises from variations in threshold voltage, mobility, and other process parameters. These mismatches result in higher input offset voltages, as observed in simulation and practical designs. In contrast, the proposed Class-AB amplifier employs a complementary input stage comprising both PMOS and NMOS transistors. This complementary architecture leverages the averaging effect of mismatch between the two transistor types, leading to significantly reduced input offset voltage. This makes the proposed design more suitable for precision applications, where low input offset and high linearity are crucial. The reduction in offset voltage is particularly evident in Monte Carlo (schematic view) simulations, which account for device-level variations. Figure 9 shows comparisons of thee designs. Other critical parameters are summarized in Table 5, including the mean (μ) and standard deviation (σ), demonstrating robustness of the proposed design against process and mismatch variation.

A detailed PVT analysis is summarized in Table 6, covering process corners (NMOS, PMOS, resistors, capacitors), supply voltage variations (±10%), and temperatures ranging from −20 °C to 80 °C, validating the reliability of the design under real-world conditions.

While the design is optimized for motion sensors with wake-up demand, Table 7 summarizes a comparison of the proposed work for various applications. 

FOM_1_ and FOM_2_ are defined as follows [3]:

*FOM*_1_ = (*SR*·*C_L_*)/(*Power*·*Area*), (10)

*FOM*_2_ = (*f_u_*·*C_L_*)/(*Power*·*Area*), (11)

where the higher the FOM, the better. The proposed design achieves ultra-low power and the smallest form factor compared to earlier work while achieving the second-best FOM_1_, FOM_2_ compared to [3] due to the longer transistor length in the CMOS technology node. As discussed earlier, the phase margin across corners for the proposed Class-AB is better than 75°, which suggests the possibility of improving the unity gain frequency as well as the slew rate and hence FOM_s_ by reducing C_c1_ and C_c2_. 

## 5. Conclusions

This work presents a compact and energy-efficient Class-AB operational amplifier tailored for motion sensor applications with wake-up functionality. By addressing the limitations of free Class-AB designs, the proposed architecture significantly reduces the inrush current from 1 mA to 7 µA, decreases the layout area by 53%, and achieves a high gain of 60 dB with a 5 pF load. The amplifier operates at a low power consumption of 2 µW, ensuring compatibility with battery-powered systems. Additionally, the design achieves an SNDR of 22 dB, meeting the stringent requirements of motion sensor interfaces for IoT and portable devices.

These advancements demonstrate the potential of the proposed design to enhance the efficiency and reliability of wake-up systems. The integration of a low inrush current biasing scheme and a compact layout makes this amplifier well-suited for resource-constrained applications such as MEMS-based accelerometers and wearable motion sensors. Furthermore, the versatile architecture of the proposed operational amplifier can be redesigned and adapted for other applications, including wireless communication standards such as Wireless Local Area Network (WLAN) and Bluetooth, with potential trade-offs in power and bandwidth.

## Figures and Tables

**Table 1:** Specifications for motion sensor analog front-end circuits across corners.

Specification [Units]	Requirement
Bandwidth [kHz]	≥10
Unity gain frequency [kHz]	≥200
Phase margin [°]	≥50
Input signal [mV]	0.5–10
ADC input full-scale [Vpp]	0.5
Integrated input-referred noise [μVrms]	≤50
Max gain [dB]	51
CMRR@10 Hz [dB]	≥40
PSRR@10 Hz [dB]	≥40
Power consumption [μW]	≤2
Slew rate [V/μs]	≥0.3
C_L_ [pF] (ADC load, pads etc.)	5
C_IN_ [pF] (Amp load, pads etc.)	≤1
Inrush current [μA]	≤10
IM3, IM2 at 1 Vpp [dBc]	≤−21 (each)
SNDR [dB]	≥20

**Table 2:** Design parameters for the three op-amps.

Class-A	Free Class-AB	Proposed Class-AB
(W/L)_7_ = 2(W/L)_3_ = 2(W/L)_4_ = 4(6 μm/0.18 μm)	(W/L)_7_ = 2(W/L)_3_ = 2(W/L)_4_ = 4(6 μm/0.18 μm)	(W/L)_12_ = 2(W/L)_10_ = 4(W/L)_5_ = 4(W/L)_6_ = 4(6 μm/0.18 μm)
(W/L)_6_ =	(W/L)_6_ =	(W/L)_11_ =
(W/L)_5_ = 4(W/L)_8_ = 4(6 μm/0.18 μm)	(W/L)_5_ = 4(W/L)_8_ = 4(6 μm/0.18 μm)	4(W/L)_7_ = 4(W/L)_8_ = 2(W/L)_9_ = 4(W/L)_14_ = 4(W/L)_15_ = 4(6 μm/0.18 μm)
(W/L)_1_ =	(W/L)_1_ =	(W/L)_1_ =
(W/L)_2_ =	(W/L)_2_ =	(W/L)_2_ =
2(6 μm/0.18 μm)	2(6 μm/0.18 μm)	(W/L)_3_ = (W/L)_4_ = 6 μm/0.18 μm
C_c_ = 1.5 pF	C_c_ = 1.5 pF	C_c1_ = 0.75 pF
$R_{c}=100\;k\Omega$	$R_{c}=100\;k\Omega$	$Rc_{1}=100\;k\Omega$
	C_B_ = 3 pF	C_c2_ = 0.75 pF
	R_B_: 1.5 μm/0.18 μm (MOSFET)	$Rc_{2}=100\;k\Omega$

**Table 3:** Small-signal simulation results for the three op-amps under typical conditions.

Specification [Units]	Class-A	Free Class-AB	Proposed Class-AB
DC small-signal open-loop gain [dB]	53	53	59
Unity gain frequency [kHz]	435.8	556.3	550.1
Phase margin [°]	57.4	78.8	82.5
Closed loop gain −3 dB BW [kHz]	664	683.5	631.7
Total integrated input noise [μVrms]	44.4	44.3	45.3
Power consumption * [μW]	1.95	1.95	2.05
Active area [μm^2^]	1568	3312	1568
PSRR@100 Hz/PSRR worst [dB]	53.6/1.4	46.6/8.3	46.2/8.5
PSRR BW [kHz]	1.54	37.3	34.3
CMRR@100 Hz/CMRR worst [dB]	45.9/30.6	49.9	52.0/37.9
CMRR BW [kHz]	39.8	54.5	52.3

* Core power.

**Table 4:** Large signal results for the three op-amps.

Specification [Units]	Class-A	Free Class-AB	Proposed Class-AB
Closed-loop gain 3 dB BW [kHz]	80	175	175
Worst IM3 within 3 dB BW [dBc]	−20.7	−20.7	−22.4
Worst IM2 within 3 dB BW [dBc]	−12.4	−29.5	−43.3
Worst slew rate [V/μs]	0.08	0.24	0.33
Steady-state error @ 300 μs [mV]	0.1	15	0.1
Inrush current [μA]	1	7	1004
Estimated SNDR at 1 Vpp operation [dB]	11.9	20.1	22.3

**Table 5:** Monte Carlo 200 pt simulation of critical parameters.

Specification [Units]	Class-A μ/σ	Free Class-AB μ/σ	Proposed Class-AB μ/σ
Input referred offset voltage [mV]	0.16/0.12	0.16/0.12	0.07/0.01
Phase margin [°]	47.3/1.5	74/1.8	78.8/3.2
DC gain [dB]	58.8/1.6	62.6/2	64.9/1.6
Unity gain frequency [kHz]	733.4/35.9	1082/79.8	1120/107.4
IM3 @ 50 kHz [dBc]	−25/0.97	−58.24	−60.55/0.78
IM2 @ 50 kHz [dBc]	−20.2/1.1	−59.2/0.67	−80.8/5.8
CMRR@100 Hz [dB]	51.8/1.6	51.7/1.6	54.9/1.6

**Table 6:** Simulated specifications at different process, voltage, and temperate corners.

Specification [Units] (T = 27 °C, VDD = 1.8 V)	TTTT			SSSS			FFFF		
	Standard	Free	Proposed	Standard	Free	Proposed	Standard	Free	Proposed
	Class-A	Class-AB	Class-AB	Class-A	Class-AB	Class-AB	Class-A	Class-AB	Class-AB
DC gain [dB]	53.0	53.0	59.0	59.5	59.5	64.8	48.4	48.4	54.8
Unity gain frequency [MHz]	0.44	0.56	0.55	0.30	0.48	0.49	0.51	0.68	0.67
Phase margin [°]	57.4	78.8	82.5	53.6	75.1	75.3	60.7	83.8	91.7
Power [μW]	1.95	1.95	2.05	1.75	1.75	1.90	2.20	2.20	2.70
PSRR@100 Hz [dB]	53.6	46.6	46.2	48.4	46.6	46.2	60.9	46.6	46.2
CMRR@100 Hz [dB]	46.9	49.9	52.0	45.1	49.9	52.0	49.9	49.9	52.0
Worst slew rate (SR) [V/μs]	0.08	0.24	0.33	0.08	0.23	0.27	0.10	0.26	0.38
IM3 @ 50 kHz [dBc]	−23.4	−53.4	−58.1	−21.4	−51.3	−53.3	−26.9	−56.9	−59.9
IM2 @ 50 kHz [dBc]	−17.7	−53.9	−69.7	−15.4	−53.4	−75.6	−20.7	−55.1	−89.7
Integrated noise [μVrms]	44.4	44.3	45.3	43.8	43.8	43.5	45.1	45.2	47.3
**Specification [Units]** **(TTTT, T = 27 °C)**	**VDD = 1.62 V**			**VDD = 1.8 V**			**VDD = 1.98 V**		
	**Standard**	**Free**	**Proposed**	**Standard**	**Free**	**Proposed**	**Standard**	**Free**	**Proposed**
	**Class-A**	**Class-AB**	**Class-AB**	**Class-A**	**Class-AB**	**Class-AB**	**Class-A**	**Class-AB**	**Class-AB**
DC gain [dB]	52.8	52.8	58.8	53.0	53.0	59.0	53.1	53.2	59.3
Unity gain frequency [MHz]	0.41	0.52	0.49	0.44	0.56	0.55	0.46	0.59	0.61
Phase margin [°]	56.3	77.6	79.3	57.4	78.8	82.5	58.4	80.0	85.8
Power [μW]	1.66	1.67	1.79	1.95	1.95	2.05	2.26	2.26	2.39
PSRR@100 Hz [dB]	54.1	46.8	46.3	53.6	46.6	46.2	53.1	46.33	46.1
CMRR@100 Hz [dB]	46.7	49.7	49.6	46.9	49.9	52.0	47.2	50.2	51.9
Worst slew rate (SR) [V/μs]	0.08	0.23	0.29	0.08	0.24	0.33	0.09	0.26	0.35
IM3 @ 50 kHz [dBc]	−22.9	−51.2	−52.9	−23.4	−53.4	−58.1	−24.0	−55.3	−58.7
IM2 @ 50 kHz [dBc]	−17.0	−52.8	−77.7	−17.7	−53.9	−69.7	−18.5	−55.0	−86.2
Integrated noise [μVrms]	44.5	44.4	45.4	44.4	44.3	45.3	44.4	44.3	45.3
**Specification [Units]** **(TTTT, VDD = 1.8 V)**	**T = −20 °C**			**T = 27 °C**			**T = 80 °C**		
	**Standard**	**Free**	**Proposed**	**Standard**	**Free**	**Proposed**	**Standard**	**Free**	**Proposed**
	**Class-A**	**Class-AB**	**Class-AB**	**Class-A**	**Class-AB**	**Class-AB**	**Class-A**	**Class-AB**	**Class-AB**
DC gain [dB]	54.0	56.6	60.3	53.0	53.0	59.0	51.8	51.8	57.7
Unity gain frequency [MHz]	0.49	0.64	0.60	0.44	0.56	0.55	0.39	0.49	0.51
Phase margin [°]	62.2	84.0	85.3	57.4	78.8	82.5	53.0	74.3	80.4
Power [μW]	1.88	1.88	2.04	1.95	1.95	2.05	2.03	2.04	2.34
PSRR@100 Hz [dB]	53.2	46.3	47.9	53.6	46.6	46.2	41.7	50.7	45.3
CMRR@100 Hz [dB]	48.6	52.7	54.4	46.9	49.9	52.0	45.2	45.2	52.6
Worst slew rate (SR) [V/μs]	0.80	0.24	0.30	0.08	0.24	0.33	0.83	0.26	0.34
IM3 @ 50 kHz [dBc]	−22.7	−52.3	−54.4	−23.4	−53.4	−58.1	−24.0	−54.5	−57.6
IM2 @ 50 kHz [dBc]	−18.3	−53.5	−78.1	−17.7	−53.9	−69.7	−17.7	−54.5	−83.9
Integrated noise [μVrms]	41.4	41.3	42.5	44.4	44.3	45.3	47.7	47.5	48.5

**Table 7:** Comparison with the state-of-the-art.

Specification [Units]	Proposed Work	[3] (2021)	[4] (2018)	[5] (2017)	[6] (2019)	[15] (2023)
CMOS process [μm]	0.18	0.13	0.18	0.35	0.18	0.04
Verification	Simulation	Measured	Measured	Measured	Measured	Simulation
Supply [V]	1.8	±0.6	±0.9	0.9	1.8	0.5
Capacitive load (*C_L_*) [pF]	5	300	25	10	5	50
Slew rate (SR) [V/μs]	0.33	5.4	28	0.25	13.25	0.055
DC gain [dB]	59	87.8	90.8	65	105.5	89
$\mathrm{Input}\;\mathrm{referred}\;\mathrm{noise}\;[\mathrm{nV}/\sqrt{Hz}]$	184.7@10 kHz	20@1 MHz	27@1 MHz	65@100 kHz	194,224@1 Hz–10 MHz	245@1 kHz
Phase margin [°]	82.5	54	58.4	65	53	63.6
Power [μW]	2.05	126	80	24.9	850	0.72
Inrush current [μA]	7	NA	NA	NA	NA	NA
Active area [mm^2^]	0.0016	0.025	0.021	1,568	0.45	NA
PSRR@10 Hz [dB]	59.6	86	64	50	NA	79
CMRR@10 Hz [dB]	59.2	92	68	80	NA	101
Unity gain frequency (*f_u_*) [MHz]	0.55	10	12.5	1	231.7	0.174
${\mathrm{FOM}}_{1}\;[V\cdot \mathrm{pF}/\mu s\cdot$μW·mm^2^]	503	514	419	7.2	0.17	NA
${\mathrm{FOM}}_{2}\;[\mathrm{MHz}\cdot \mathrm{pF}/$μW·mm^2^]	838.4	952.4	186	28.7	3	NA

## Data Availability

The data are contained within the article.
